# Association between Blood Mercury Levels and Non-Alcoholic Fatty Liver Disease in Non-Obese Populations: The Korean National Environmental Health Survey (KoNEHS) 2012–2014

**DOI:** 10.3390/ijerph18126412

**Published:** 2021-06-13

**Authors:** Yun-Jung Yang, Eun-Jung Yang, Kyongjin Park, Subin Oh, Taehyen Kim, Yeon-Pyo Hong

**Affiliations:** 1 Institute of Biomedical Science, Catholic Kwandong University International St. Mary’s Hospital, Incheon 22711, Korea; yangyj@ish.ac.kr; 2 Department of Plastic and Reconstructive Surgery, Yonsei University College of Medicine, Seoul 03722, Korea; enyang7@yuhs.ac; 3 College of Medicine, Catholic Kwandong University, Gangneung-si 25601, Korea; kyongjin99@gmail.com (K.P.); rosette731@gmail.com (S.O.); starkim3@naver.com (T.K.); 4 Department of Preventive Medicine, College of Medicine, Chung-Ang University, Seoul 06974, Korea

**Keywords:** mercury, hepatic steatosis index, non-alcoholic fatty liver disease, Korean National Environmental Health Survey, non-obese

## Abstract

Mercury is widely distributed in the environment, and a plausible association between mercury exposure and hepatic damage has been reported. Non-alcoholic fatty liver disease (NAFLD), which comprises a spectrum of liver diseases, has recently been recognized in non-obese subjects. However, there have been no studies on the relationship between internal mercury levels and NAFLD in non-obese individuals. Therefore, we investigated the association between blood mercury levels and NAFLD in non-obese subjects. Cross-sectional data (*n* = 5919) were obtained from the Korean National Environmental Health Survey (2012–2014). NAFLD was defined using the hepatic steatosis index (HSI). Blood mercury levels were log-transformed and divided into quartiles based on a weighted sample distribution. The association between blood mercury levels and NAFLD was analyzed using a multivariate logistic analysis after body mass index stratification. The geometric mean of blood mercury in the overweight group was significantly higher than that of the non-obese group (*p* < 0.001). The weighted frequencies of patients with NAFLD based on the HSI were 3.0–7.2% for the non-obese subjects and 52.3–63.2% for the overweight subjects. In the multivariate analysis, blood mercury levels were positively associated with NAFLD for both the overweight and non-obese groups (all *p* for trend < 0.001). Increased blood mercury levels are closely associated with NAFLD. In particular, mercury could be a risk factor for NAFLD in the non-obese population.

## 1. Introduction

Mercury is present in the environment in the form of elemental (metallic) mercury, inorganic mercury compounds, and organic mercury [1,2]. Because elemental mercury is liquid at room temperature, it can be easily released into the atmosphere. Inorganic mercury combines readily with chlorine, sulfur, and oxygen and is deposited in water and soil as inorganic mercury salts. Organic mercury is formed by combining methylmercury and carbon. Methylmercury, which is highly toxic, is the most widely found form of organic mercury in the environment. 

Humans are exposed to elemental mercury through the respiratory tract, inorganic mercury from food, and methylmercury from seafood [3]. The half-life of mercury in the blood is approximately 2 months [4], and it is estimated to be as long as 20 years in the brain [5]. The geometric means of blood mercury levels were 3.12 μg/L [6] and 3.80 μg/L [7], in Korea, which are approximately four to six times greater than those in the US and European countries [8,9]. Once internalized, mercury travels to various organs through the bloodstream, and it may increase oxidative stress, thereby increasing the adverse effects in the nervous, endocrine, and reproductive systems [1,10,11]. Recently, it has been reported that the elevation of oxidative stress and alteration of mitochondrial functions may also increase the risk of metabolic disorders as well as liver dysfunctions [12,13]. 

Liver enzyme levels, which are a proxy marker for liver dysfunction and non-alcoholic fatty liver disease (NAFLD), were found to be associated with mercury exposure in recent epidemiological studies [14,15,16]. NAFLD may progress to liver failure and hepatocellular carcinoma [17]; accordingly, epidemiological interest in the search for factors related to the occurrence of NAFLD is increasing.

The prevalence of NAFLD, which is the most common form of chronic liver disease, is constantly increasing worldwide [18,19]. Although the onset of NAFLD is closely associated with obesity [20], it also occurs in non-obese people [21,22]. The prevalence of NAFLD in the non-obese population is about 5–26% [23]. Most non-obese NAFLD patients have visceral fat, which is related to insulin resistance (IR) [22,24]. The relationship between internal mercury levels and IR has been investigated in a non-diabetic population [25]; however, no studies have reported on an association between blood mercury and non-obese NAFLD.

Therefore, the purpose of this study was to evaluate the association between mercury concentration and the risk of NAFLD in overweight and non-obese individuals after stratification based on body mass index (BMI). 

## 2. Materials and Methods

### 2.1. Study Population

A total of 6478 participants (≥19 years old) were studied based on data extracted from the second Korean National Environmental Health Survey (KoNEHS, 2012–2014). This survey is conducted every 3 years with the objective of identifying human exposure to environmental risk factors and to follow changes to the spatiotemporal distribution among the Korean population. In the second KoNEHS, the study group was sorted using a multistage stratified cluster sampling method. Anthropometric data and blood and urine samples were collected through face-to-face encounters with each participant. All the participants provided written informed consent. Blood and urine samples were collected for an analysis of various clinical values and environmental chemicals. 

**Figure 1:**
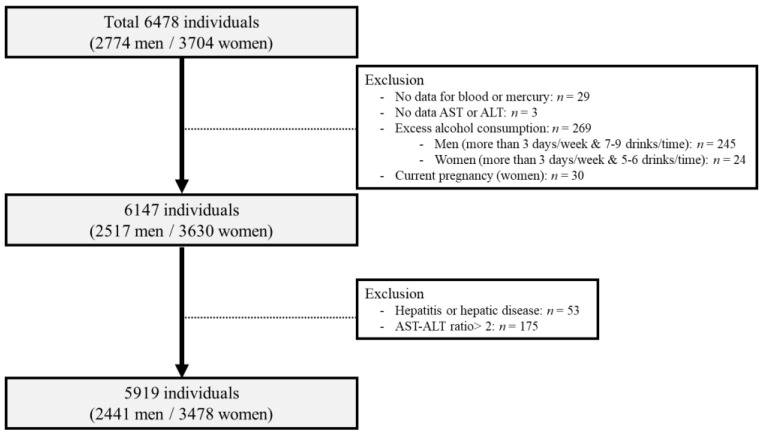
Flow diagram of study participants in the study obtained from the Korean National Environmental Health Survey II (2012–2014).

We excluded participants who had any of the following conditions at baseline (Figure 1): no data on blood mercury levels (*n* = 29); no data on alanine aminotransferase (ALT) or aspartate aminotransferase (AST) levels (*n* = 3); a history of liver disease, such as hepatitis or hepatic cirrhosis (*n* = 53); an AST/ALT ratio > 2 (*n* = 175); significant alcohol consumption (more than 3 days per week and ≥7–9 drinks per time for men [*n* = 245] or more than 3 days per week and ≥5–6 drinks per time for women [*n* = 24]); and currently pregnant (*n* = 30). A total of 5919 participants were finally included in the study (men: *n* = 2441 and women: *n* = 3478).

### 2.2. Questionnaire and Definition of Anthrophometric and Biochemical Parameters

The general characteristics of the participants including age, gender, alcohol consumption, smoking status, physical activity, monthly household income, education, and marital status were obtained from face-to-face interviews using a questionnaire. The general characteristic subcategories were as follows: drinking and smoking status (current, past, and never); physical activity (vigorous, moderate, and none); monthly household income (low, low-mid, mid-high, and high); and marital status (single, married, and divorced). 

Hepatic disease was defined as a self-reported history of diagnosed hepatitis or fatty liver disease and currently undergoing treatment or taking medication. Participants who had hypertension or were taking antihypertensive drugs were classified as hypertensive. Diabetes mellitus (DM) was defined as a self-reported history of DM or the use of antidiabetic drugs. Hyperlipidemia was defined as a self-reported history of hyperlipidemia, use of anti-hyperlipidemia drugs, a triglyceride (TG) level ≥ 240 mg/dL, or a high-density lipoprotein cholesterol level ≤ 40 mg/dL. Data on serum TG, ALT, and AST levels were also obtained. 

BMI was calculated by dividing each participant’s body weight (kg) by their height squared (m^2^). The expected ALT and AST values were 10–49 U/L and <34 U/L, respectively. In addition, the GGT reference ranges were <73 U/L for men and <38 U/L for women. Abnormal ALT, AST, and GGT activities were defined as values outside the reference range.

### 2.3. NAFLD Assessment 

The presence of NAFLD was determined using the hepatic steatosis index (HSI), which is relatively an effective and non-invasive NAFLD detection marker [26]. The HSI was calculated using the formula HSI = 8 × ([ALT]/[AST] ratio) + BMI (+2, if female; +2, if DM). HSI values ≥ 36 were defined as indicating NAFLD, and HSI values < 36 were defined as non-NAFLD [26]. 

### 2.4. Statistical Analysis

The participants were divided into two groups according to their BMIs. Participants with BMI < 25 kg/m^2^ were assigned to the non-obese group and those with BMI ≥ 25 kg/m^2^ were assigned to the overweight group, according to classifications developed by the World Health Organization. Blood mercury levels were log-transformed due to right skewness. The average (continuous variables) and frequency (categorical variables) were compared using a T-test or Chi^2^ test, respectively. Blood mercury levels were divided into quartiles based on the weighted sample distribution. The lowest quartile was used as a reference. The relationship between blood mercury levels and HSI was assessed using a multivariate logistic regression. Multivariate analysis included demographic characteristics and clinical variables. Model 1 was adjusted for non-modifiable risk factors such as age and sex. Model 2 was additionally adjusted for well-known general characteristics such as smoking, drinking, exercise, marital status, education, and income. Model 3 was additionally adjusted for proven modifiable risk factors such as hypertension, diabetes mellitus, hyperlipidemia, and seafood consumption. The data were analyzed using STATA version 16.0 (StataCorp LP， College Station, TX, USA). Statistical significance was set at *p* < 0.05.

## 3. Results

### 3.1. General Characteristics

The general characteristics of the participants, including age, sex, drinking, smoking, physical activity, monthly household income, education, and marital status are shown for the non-obese and overweight groups in Table 1. The proportion of men and the average age were significantly higher in the overweight group than in the non-obese group (all *p* < 0.001). Drinking status and physical activity were not significantly different between the two groups (*p* = 0.151 and *p* = 0.596, respectively). Smoking status, monthly household income, education level, and marital status were significantly different (all *p* < 0.001); however, the proportions of hypertension, DM, and hyperlipidemia were significantly higher in the overweight group than in the non-obese group (all *p* < 0.001). 

### 3.2. Mercury Concentration in Blood and Urine

The blood mercury concentrations in the non-obese and overweight groups are shown in Table 2. The ranges of blood mercury in the non-obese and overweight groups were 0.07–62.74 μg/L and 0.50–115.62 μg/L, respectively. The blood mercury of the overweight group was statistically higher than that of the non-obese group (*p* < 0.001). 

### 3.3. Prevalence of NAFLD

The prevalence of NAFLD in the non-obese and overweight groups was evaluated based on the HSI and abnormal AST, ALT, and GGT levels (Table 3). The overall weighted frequencies (%) of patients with NAFLD based on the HSI was 16.33% in the lowest quartile, and it increased with increasing blood mercury levels to 31.63% in the highest quartile (*p* < 0.001). The weighted frequencies (%) of NAFLD based on the abnormal AST, ALT, and GGT levels also increased with increasing blood mercury levels (all *p* < 0.001). 

In the non-obese group, the weighted frequencies (%) of patients with NAFLD based on the HSI was 2.98% in the lowest quartile of blood mercury levels, and it increased with increasing blood mercury levels to 7.15% in the fourth quartile (*p* < 0.001). The prevalence of elevated ALT, AST, and GGT was significantly increased according to increasing blood mercury levels (*p* = 0.020, *p* < 0.001, and *p* = 0.020, respectively). 

The weighted frequencies (%) ranged from 52.34% to 63.18% in the overweight group, but the difference was not statistically significant (*p* = 0.523). The weighted frequencies (%) of the abnormal ALT, AST, and GGT levels increased significantly with increasing blood mercury concentrations (*p* = 0.003, *p* = 0.022, and *p* = 0.003, respectively). 

### 3.4. Association between Mercury and NAFLD

**Figure 2:**
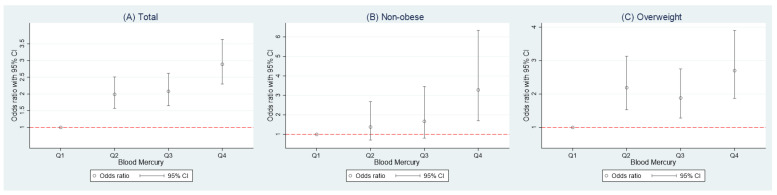
Multivariate odds ratio and 95% confidence intervals (CI) for NAFLD (**A**) Total, (**B**) Non-obese, and (**C**) Overweight according to the quartile of blood mercury levels. Adjusted for age, sex, smoking, drinking, exercise, marital status, education, income, hypertension, diabetes mellitus, hyperlipidemia, and seafood consumption within one week. Non-obese: body mass index (BMI) ≤ 25 kg/m^2^, Overweight: BMI > 25 kg/m^2^.

A logistic regression analysis was performed to investigate the association between the HSI and blood mercury concentration (Table 4). The lowest quartile of the blood mercury concentration (first quartile) was used as a reference. The crude and multivariate analyses for the overall group showed that higher blood mercury levels were significantly associated with a progressively higher NAFLD OR (Figure 2A). 

In the non-obese group, the fourth quartile of the blood mercury levels had significantly higher ORs than the lowest quartile (OR: 2.50, 95% confidence interval [CI]: 1.33–4.72). After adjusting for age and sex, the adjusted OR (95% CI) in the fourth quartile compared to the first quartile was 2.23 (1.16–4.30). When adjusted additionally for smoking, drinking, physical activity, marital status, education level, and monthly household income, the fourth quartile showed a significantly higher OR than the first quartile (OR: 2.68, 95% CI 1.39–5.14). Further adjustments were performed with clinical variables including hypertension, DM, hyperlipidemia, and seafood consumption within one week. The OR (95% CI) in the fourth quartile with these adjustments was 3.28 (1.69–6.35) compared to the reference category (Figure 2B). 

The overweight group showed the significant differences in the second and fourth quartiles compared with those in the lowest quartile (OR: 1.56, 95% CI: 1.13–2.14, and OR: 1.41, 95% CI 1.03–1.94, respectively). In the age- and sex-adjusted model, the ORs from the second to fourth quartiles were significantly higher than the lowest quartile of the reference category (*p* for trend = 0.001). After additional adjustments for demographic factors and clinical variables (model 2 and 3), the ORs did not change significantly when compared to the univariate estimate (all *p* for trend < 0.001) (Figure 2C).

## 4. Discussion

In this study, we investigated the association between blood mercury concentrations and NAFLD. The study subjects were classified into non-obese and overweight groups according to their BMIs (<25.0 kg/m^2^ and ≥25 kg/m^2^, respectively). The weighted frequencies of patients with NAFLD based on the HSI scores in the non-obese group were lower than those of the overweight group. High blood mercury levels were associated with NAFLD in both the non-obese and overweight groups. After adjusting for the covariates, blood mercury levels were significantly associated with NAFLD in the overweight subjects. In the non-obese group, the highest blood mercury levels were associated with NAFLD in both univariate and multivariate analyses. 

There are three forms of mercury in the environment: elemental (or metallic), inorganic, and organic mercury (e.g., methylmercury) [1]. In the sea and rivers, inorganic mercury is biotransformed by microbial activity into organic forms, which then accumulate in seafood [3]. Although inhalation of mercury vapors is a potential source of exposure in the general population, dietary intake through fish and other seafood consumption is the predominant source of mercury exposure [1]. The administered mercury following fish and seafood intake is absorbed into the bloodstream, and moved to the liver [27]. Mercury levels in the blood are highest at within 10 h after digestion, and the disappearance of mercury from blood is biphasic with average half-times of 7.7 h and 52 days, respectively [4,28]. The metabolized mercury is absorbed across the intestine and excreted in feces [27], thereby blood mercury levels are considered a more appropriate biomarker than urine. 

A positive association between blood mercury levels and obesity in the adult population is reported [29,30]. Experimental studies have suggested that higher levels of mercury are detected in the blood and other organs of obese animals compared with the normal-weight animals [31,32]. Although mercury could increase the risk of the occurrence of obesity-related metabolic disorders, the non-significant result [33] and negative association [34] were observed in the relationship between blood mercury levels and central obesity.

Exposure to mercury increases oxidative stress and decreases antioxidant levels, subsequently inducing organ damage [10,35]. In epidemiological studies, internal mercury levels are positively associated with oxidative marker levels [11,36]. The imbalance between the production of reactive oxygen species and the capacity of the antioxidant system caused by mercury exposure may affect the development of metabolic diseases, including insulin resistance, obesity, type 2 DM, and hypertension [12]. In addition, mercury-induced liver damages might be linked to mitochondrial degeneration and an increase in oxidative stress in the endoplasmic reticulum [12,13]. Nevertheless, as obesity is considered a primary factor of NAFLD [20], mercury could lead to an increased risk of NAFLD occurrence and progression regardless of the obesity. 

NAFLD is defined as ≥5% steatosis without significant alcohol consumption or competing liver diseases [37]. The spectrum includes diseases ranging from simple steatosis to non-alcoholic steatohepatitis, advanced fibrosis, hepatocellular carcinoma, and liver failure [17]. A positive association between blood mercury levels and the risk of NAFLD (based on ALT levels) has been reported in both US adolescents and adults [14,15]. In addition, blood mercury levels in the men were shown to be significantly associated with NAFLD, but not in the women, after adjusting for comorbidities [16]. These studies used BMI as a covariate in the multivariate analyses, instead of stratification. 

In the non-obese Asians, higher BMIs, a homeostatic model assessment of insulin resistance values, ALT levels, hypertriglyceridemia, and hyperuricemia were associated with the presence of NAFLD [22,38]. This suggests that an increase in visceral fat accumulation and the waist-hip ratio influences the development of non-obese NAFLD. In this study, the highest quartile of blood mercury concentration significantly increased the risk of NAFLD compared to the lowest quartile in the non-obese population. Blood mercury levels in the overweight subjects were also closely associated with NAFLD. A longitudinal study is required to clarify the relationships between blood mercury and NAFLD in both non-obese and overweight populations. 

Biochemical parameters, including ALT, AST, and GGT, are useful biomarkers to identify the liver damage. The unexplained increase in ALT by viral hepatitis, ethanol, or iron overload might indicate the possibility of the presence of NAFLD [38,39]. The suspected NAFLD using unexplained elevated ALT was 5.4% in US adults [39] and 8.7% in Taiwan adults [38]. In middle age (50–59 years), the elevation of ALT and AST was associated with BMI [39]. In addition, because the inverse relationship between serum GGT and antioxidants was already reported, serum GGT might be an early marker of oxidative stress [40]. It has been reported that mercury is significantly associated with the elevation of ALT, AST, and GGT [16,40,41]. In this study, the prevalence of abnormal ALT, AST, and GGT levels increased according to blood mercury levels. It seems that the serum levels of these liver enzymes could detect liver dysfunctions which are possibly linked to NAFLD. 

This study, to the best of our knowledge, is the first to discover a positive relationship between blood mercury levels and NAFLD in a non-obese population. Additionally, the study population included a large and uniform sample population. Nevertheless, several limitations should be considered. First, NAFLD was defined using HSI scores. Although a liver biopsy and abdominal ultrasound are the most definitive NAFLD diagnostic methods, they are difficult to use in population-based studies because of the cost-effectiveness. HSI had an area under the receiver-operating curve of 0.812 (95% CI 0.801–0.824), with a sensitivity of 93.1% and a specificity of 92.4% [28]. In the equation, two points were added to females to adjust for the difference in BMI between male and female individuals. Thus, the use of HSI might be utilized to predict the presence of NAFLD in large-scale studies. Nevertheless, there might be a classification error, because the intermediate group (30 ≤ HSI ≤ 36) was included in the non-NAFLD group. However, it was inevitable for statistical analysis. Second, the overweight and non-obese individuals were classified by BMI. Because BMI does not reflect the amount of visceral fat, the waist-hip ratio or waist circumstance should be considered to clarify the association between mercury exposure and non-obese NAFLD. Third, we could not evaluate the amount of alcohol consumption (g/day) because KoNEHS offered the drinking times in the last month and the number of glass per times. In this study, men who consumed alcohol three times or more in a week and 7–9 glasses per time, and women who consumed alcohol three times or more in a week and 5–6 glasses per time were defined as heavy drinkers by referring to a previous study [42]. Fourth, there might be a lack of information such as the history of medications (including estrogen) and the history of viral hepatitis or another hepatic disease. Those items are included in the questionnaire; however, they may not have been accurately investigated due to recall bias. Further studies providing data on intake of medication and hepatic disease are needed to confirm our results. Last but not least, the results could not estimate the causal relationship between mercury exposure and NAFLD due to the cross-sectional nature of the study. A longitudinal study is needed to determine the contribution of mercury on NAFLD prevalence. 

## 5. Conclusions

The results of this study demonstrate that blood mercury levels are closely associated with NAFLD. Although the number of non-obese NAFLD patients was lower than that of the overweight NAFLD patients, mercury might still elevate the risk of NAFLD, particularly in the low- and normal-weight population. Further mechanistic studies are needed to determine whether mercury induces liver damage and NAFLD in both non-obese and overweight populations. 

## Figures and Tables

**Table 1:** General characteristics of study participants according body mass index.

	Total (*n* = 5919)	Non-Obese (*n* = 3614)	Overweight (*n* = 2305)	*p*-Value
Gender, *n* (% men)	2441 (41.24)	1409 (38.99)	1032 (44.77)	<0.001
Age (years)	51.37 ± 0.19	49.89 ± 0.25	53.70 ± 0.30	<0.001
Drinking Status, *n* (%)				0.151
Never	2121 (35.83)	1304 (36.08)	817 (35.44)	
Former	315 (5.32)	176 (4.87)	139 (6.03)	
Current	3483 (58.84)	2134 (59.05)	1349 (58.52)	
Smoking Status, *n* (%)				<0.001
Never	4005 (67.66)	2510 (69.45)	1495 (64.86)	
Former	910 (15.37)	508 (14.06)	402 (17.44)	
Current	1004 (16.96)	596 (16.49)	408 (17.70)	
Physical activity, *n* (%)				0.596
No	3772 (63.73)	2320 (64.19)	1452 (62.99)	
Moderate	1169 (19.75)	709 (19.62)	460 (19.96)	
Vigorous	978 (16.52)	585 (16.19)	393 (17.05)	
Monthly household income, *n* (%)				<0.001
<Low	1632 (27.57)	901 (24.93)	731 (31.71)	
Low-Mid	2722 (45.99)	1716 (47.48)	1006 (43.64)	
Mid-High	1512 (25.54)	964 (26.67)	548 (23.77)	
>High	53 (0.90)	33 (0.91)	20 (0.87)	
Education, *n* (%)				<0.001
<High school	2115 (35.95)	1100 (30.87)	1006 (43.93)	
High school	2025 (34.42)	1290 (35.90)	735 (32.10)	
College and more	1743 (29.63)	1194 (33.23)	549 (23.97)	
Marital status, *n* (%)				<0.001
Single	610 (10.31)	441 (12.20)	169 (7.33)	
Married	4675 (78.98)	2839 (78.56)	1836 (79.65)	
Divorced	634 (10.71)	334 (9.24)	300 (10.71)	
AST	24.71 ± 0.15	23.74 ± 0.20	26.22 ± 0.23	<0.001
ALT	24.03 ± 0.22	20.95 ± 0.24	28.86 ± 0.40	<0.001
GGT	31.69 ± 0.56	27.37 ± 0.70	38.45 ± 0.92	<0.001
Comorbidity, *n* (%)				
Hypertension	1210 (20.44)	530 (14.67)	680 (29.50)	<0.001
Diabetes mellitus	493 (8.33)	218 (6.03)	275 (11.93)	<0.001
Hyperlipidemia	1925 (35.52)	924 (25.57)	1001 (43.43)	<0.001

AST: Aspartate aminotransferase; ALT: Alanine aminotransferase; GGT: Gamma glutamyl transpeptidase; HDL: High-density lipoprotein. Non-obese: body mass index (BMI) ≤ 25 kg/m^2^, Overweight: BMI > 25 kg/m^2^. Data were expressed as mean ± standard error (continuous), and number and frequency (categorical).

**Table 2:** Distributions of blood and urinary mercury levels in the study population.

Blood Mercury (ug/L)		Total (*n* = 5919)	Non-Obese (*n* = 3614)	Overweight (*n* = 2305)	*p*-Value
GM ± GSE		1.15 ± 0.01	1.08 ± 0.01	1.25 ± 0.01	<0.001
Percentile	Min	0.07	0.07	0.50	
	25th	2.05	1.93	2.28	
	50th	3.07	2.87	3.42	
	75th	4.7	4.36	5.25	
	Max	115.62	62.74	115.62	

GE: geometric mean; GSE: geometric standard error; Non-obese: body mass index (BMI) ≤ 25 kg/m^2^, Overweight: BMI > 25 kg/m^2^. Statistical analysis was performed using log-transformed blood mercury concentrations.

**Table 3:** Prevalence of NAFLD and abnormal AST, ALT, and GGT activities according to blood mercury levels in the study population.

		Quartile 1 (*n* = 1467)	Quartile 2 (*n* = 1471)	Quartile 3 (*n* = 1492)	Quartile 4 (*n* = 1489)	*p*-Value for Trend
Total						
NAFLD	Number	268	368	395	462	<0.001
	Weighted frequency (95% CI)	16.33 (14.09–18.84)	25.09 (22.08–28.36)	26.13 (23.35–29.12)	31.63 (28.73–34.69)	
Abnormal ALT	Number	103	142	139	198	<0.001
	Weighted frequency (95% CI)	8.74 (6.84–11.11)	11.46 (9.41–13.90)	11.25 (9.21–13.68)	16.32 (13.94–19.00)	
Abnormal AST	Number	92	124	121	184	<0.001
	Weighted frequency (95% CI)	5.63 (4.21–7.48)	9.60 (7.64–12.00)	8.77 (7.07–10.82)	12.94 (10.70–15.55)	
Abnormal GGT	Number	99	146	177	240	<0.001
	Weighted frequency (95% CI)	5.81 (4.28–7.84)	9.92 (8.15–12.02)	10.56 (8.78–12.65)	15.10 (13.10–17.33)	
Non-obese (*n* = 3614)						
NAFLD	Number	29	36	36	49	<0.001
	Weighted frequency (95% CI)	2.98 (1.79–4.91)	3.41 (2.21–5.21)	4.17 (2.62–6.57)	7.15 (5.13–9.89)	
Abnormal ALT	Number	51	55	45	63	0.020
	Weighted frequency (95% CI)	6.46 (4.59–9.01)	6.92 (5.00–9.49)	6.37 (4.28–9.36)	9.19 (6.67–12.53)	
Abnormal AST	Number	49	49	51	74	<0.001
	Weighted frequency (95% CI)	4.51 (3.02–6.67)	5.63 (3.79–8.30)	5.83 (3.99–8.46)	8.77 (6.39–11.92)	
Abnormal GGT	Number	51	62	73	96	0.020
	Weighted frequency (95% CI)	4.46 (3.06–6.46)	6.22 (4.44–8.63)	6.65 (4.93–8.92)	11.51 (9.01–14.58)	
Overweight (*n* = 2305)						
NAFLD	Number	239	332	359	413	0.523
	Weighted frequency (95% CI)	52.34 (46.20–58.40)	63.18 (57.07–68.89)	58.44 (53.57–63.15)	60.91 (56.18–65.44)	
Abnormal ALT	Number	52	87	94	135	0.003
	Weighted frequency (95% CI)	14.92 (10.81–20.23)	19.45 (14.95–24.91)	18.44 (14.66–22.93)	24.84 (20.69–29.52)	
Abnormal AST	Number	43	75	70	110	0.022
	Weighted frequency (95% CI)	8.65 (5.87–12.59)	16.57 (12.43–21.75)	13.09 (10.08–16.83)	17.92 (14.22–22.33)	
Abnormal GGT	Number	48	84	104	144	0.003
	Weighted frequency (95% CI)	9.44 (6.07–14.38)	16.42 (12.81–20.81)	16.32 (12.85–20.50)	19.39 (15.79–23.58)	

NAFLD: non-alcoholic fatty liver disease; HSI: Hepatic steatosis index; AST: Aspartate aminotransferase; ALT: Alanine aminotransferase; GGT: gamma glutamyl transpeptidase; CI Confidence interval; Non-obese: body mass index (BMI) ≤ 25 kg/m^2^, Overweight: BMI > 25 kg/m^2^.

**Table 4:** Association between blood mercury levels and NAFLD.

	Crude		Model 1		Model 2		Model 3	
	OR (95% CI)	*p*-Value	OR (95% CI)	*p*-Value	OR (95% CI)	*p*-Value	OR (95% CI)	*p*-Value
Total								
Quartile 1	1	<0.001 *	1	<0.001 *	1	<0.001 *	1	<0.001 *
Quartile 2	1.71 (1.37–2.15)	<0.001	1.70 (1.36–2.13)	<0.001	1.78 (1.43–2.21)	<0.001	1.99 (1.58–2.52)	<0.001
Quartile 3	1.81 (1.43–2.28)	<0.001	1.76 (1.40–2.23)	<0.001	1.91 (1.53–2.38)	<0.001	2.09 (1.66–2.64)	<0.001
Quartile 4	2.37 (1.92–2.92)	<0.001	2.27 (1.83–2.81)	<0.001	2.52 (2.04–3.11)	<0.001	2.89 (2.30–3.62)	<0.001
Non-obese (*n* = 3614)								
Quartile 1	1	0.004 *	1	0.016 *	1	0.002 *	1	<0.001 *
Quartile 2	1.14 (0.59–2.22)	0.681	1.11 (0.57–2.16)	0.738	1.24 (0.65–2.38)	0.507	1.38 (0.71–2.68)	0.330
Quartile 3	1.41 (0.68–2.91)	0.343	1.31 (0.63–2.71)	0.460	1.48 (0.74–2.97)	0.265	1.67 (0.81–3.47)	0.161
Quartile 4	2.50 (1.33–4.72)	0.005	2.23 (1.16–4.30)	0.016	2.68 (1.39–5.14)	0.003	3.28 (1.69–6.35)	<0.001
Overweight (*n* = 2305)								
Quartile 1	1	0.118 *	1	0.001 *	1	<0.001 *	1	<0.001 *
Quartile 2	1.56 (1.13–2.14)	0.006	1.68 (1.21–2.34)	0.002	1.85 (1.32–2.59)	<0.001	2.19 (1.53–3.13)	<0.001
Quartile 3	1.28 (0.92–1.77)	0.134	1.48 (1.05–2.09)	0.024	1.69 (1.18–2.42)	0.004	1.88 (1.28–2.75)	0.001
Quartile 4	1.41 (1.03–1.94)	0.030	1.86 (1.35–2.58)	<0.001	2.21 (1.56–3.11)	<0.001	2.69 (1.86–3.87)	<0.001

NAFLD: non-alcoholic fatty liver disease; OR: odds ratio; CI: confidence interval. Non-obese: body mass index (BMI) ≤ 25 kg/m^2^, Overweight: BMI > 25 kg/m^2^. *: *p* values were analyzed using the test of trend of odds. Crude: hepatic steatosis index, blood mercury level. Model 1: crude + age, sex. Model 2: model 1 + smoking, drinking, exercise, marital status, education, and income. Model 3: model 2 + hypertension, diabetes mellitus, hyperlipidemia, and seafood consumption within one week.

## Data Availability

This study used data from the Second Korean National Environmental Health Survey (KoNEHS) which was conducted by Ministry of Environment, National Institute of Environmental Research. The data presented in this study are available on request from the corresponding author. The data are not publicly available due to protect personal information.
